# Data on response of watermelon (*Citrullus lanatus* [Thunb.] Matsum. and Nakai) varieties to potassium fertilizer rates at West Dembia District, Central Gondar Zone, Ethiopia

**DOI:** 10.1016/j.dib.2025.111310

**Published:** 2025-01-23

**Authors:** Hulushum Woreta Kassa, Asrat Ayalew Teka, Aleminew Tagele Dellele, Abebaw Mulugeta Andualem

**Affiliations:** a Departmnet of Horticulture, Debre Markos University, Po Box: 269, Debre Markos, Ethiopia; b Department of Horticulture, College of Agriculture and Environmental Sciences, University of Gondar, Po Box 196, Ethiopia

**Keywords:** Dataset, Avallon F1 variety, Lahat F1 variety, Quality, Total soluble solids

## Abstract

Watermelon, a nutritious fruit, is increasingly popular in Ethiopia, but its productivity, quality, and storability are constrained by insufficient potassium (K) fertilizer and limited hybrid variety use. An experiment was conducted in Gorgora at the University of Gondarʼs research farm to evaluate the response of two hybrid watermelon varieties (Lahat F1 and Avallon F1) to five K fertilizer rates (0, 50, 100, 150, and 200 kg ha⁻¹). The results revealed that K fertilizer rates, varieties, and their interaction significantly influenced growth, fruit yield, and quality attributes. Lahat F1 treated with 150 kg K ha⁻¹ produced the highest average fruit weight (16.13 kg), marketable yield (69.60 t ha⁻¹), and fruit pulp weight (8.53 kg), achieving a marginal rate of return of 3509.23 %. These findings suggest that applying 150 kg K ha⁻¹ on Lahat F1 is optimal for watermelon production in Gorgora and similar agro-ecological zones. Further multi-seasonal and multi-location trials are recommended for broader validation.

Specifications Table

**Table:** 

Subject	Agriculture
Specific subject area	Horticulture
Type of data	Raw, Tables, Figures
Description of data collection	Data were collected from a field experiment at the University of Gondar's research farm in Gorgora, using a factorial design with two hybrid watermelon varieties (Lahat F1 and Avallon F1) and five potassium fertilizer rates (0, 50, 100, 150, and 200 kg K ha⁻¹). Crop phenology and growth parameters were measured using a measuring tape and calipers. Yield data, including fruit weight and marketable yield, were recorded with a digital weighing scale (model A&D EK-6100i). Fruit quality parameters such as pulp weight and total soluble solids were measured using a refractometer (Atago PAL-1). Data were analyzed with R software (version 4.2) using ANOVA for variance analysis, and mean comparisons were made with the least significant difference method
Data source location	The data were collected at the demonstration and research farm of the University of Gondar, located in Gorgora, Central Gondar Zone, Ethiopia. The geographical coordinates of the site are approximately 12.23° N latitude and 37.30° E longitude. The raw data are stored and managed at the Department of Horticulture, University of Gondar.
Data accessibility	Repository name: Mendeley data doi: 10.17632/scjgck7398.1 Direct URL to: https://data.mendeley.com/datasets/scjgck7398/1
Related research article	None

## 1 Value of the Data

• These data offer a comprehensive evaluation of how different potassium fertilizer rates impact hybrid watermelon varieties in Ethiopiaʼs West Dembia District. This information is crucial for agricultural researchers looking to improve nutrient management strategies and enhance crop productivity, particularly in potassium-deficient regions.
• The dataset provides a robust foundation for comparative studies and meta-analyses, enabling scientists to examine fertilizer responses across various environments. This could facilitate broader insights into optimizing potassium use for watermelons and other crops in similar agro-climatic zones.
• Researchers in precision agriculture can leverage these data to refine predictive models for crop performance based on potassium inputs, helping drive more accurate fertilizer recommendations that increase yield while reducing environmental impact.
• These data are instrumental in exploring nutrient interactions in hybrid crops, contributing to the development of sustainable farming practices that maximize crop quality and efficiency under varied growing conditions.
• This dataset can be used as a benchmark for future studies conducted in different regions and seasons, enabling the creation of location-specific fertilizer management strategies to ensure optimal crop performance.

## 2 Background

Watermelon (*Citrullus lanatus* [Thunb.] Matsum. and Nakai), a member of the Cucurbitaceae family, is cultivated for its fruit and vegetative parts (seeds and rind) and accounts for 6.8 % of the global vegetable production [1]. It contains high quantity of vitamins, minerals and antioxidant compounds such as lycopene which play important role in preventing cell damage, neutralizing and removing free radicals and help fight off different kinds of cancers [2,3]. It is a fruit that is rich in an amino acid known as l-citrulline, which the body converts to l-arginine, an essential amino acid that helps relax blood vessels and improve circulation [4]. Watermelon consists of 92 % water and it contains 7.5 g of carbohydrate in 100 g. Dietary intake of watermelon is important in maintaining human health and well-being [5]. Its global consumption is greater than that of any other cucurbits [6].

In Ethiopia, the demand for watermelon by foreigners and some of the society in large cities is increasing. Regardless of its current demand, various factors affect improvement in watermelon production in the country. The growth, yield, and quality of watermelon are influenced by various factors, including genotype, growing conditions, pests, diseases, and inadequate cultural practices. Therefore, its productivity, quality and production expansion in Ethiopia is very low compared to the suitability of the climatic and edaphic factors of the country [7].

Essential plant nutrients affect the growth, yield and quality of watermelon. These nutrients are necessary not only for crop yield but also for the maintenance of soil nutrient and quality of produce. Besides yield in terms of quantity and quality of fruit is also equally important. Fruit yield and quality of watermelon could be boosted by providing proper dose of macro and micronutrients.

Potassium (K), one of the most important essential elements helps in translocation of carbohydrates, increases disease resistance in plants and counteracts the injurious effect of nitrogen [8]. It is the second most abundant macronutrient element after nitrogen in terms of amounts found in plant tissues except seeds and plays a key regulatory role in many physiological processes vital to plant growth [9].

Nowadays, the soil status of K is significantly declined due to crop removal without compensating through K fertilizer [10]. The principal sources of K around the world are clay minerals in soils and rocks, and salt deposits in the ocean in the process of crystallization of K salts in dried sea. Inorganic fertilizers are the main sources of K [11].

Productivity and expansion of the crop could be enhanced through systematic investigation that focuses on the use of K fertilizer and hybrid variety combination to come up with concrete recommendation for increasing growth, fruit yield and quality. Therefore, the present study was to investigate the optimum rate of K for a specific watermelon hybrid variety in order to produce a satisfactory yield and quality of the plant.

## 3 Data Description

**Fig. 1:**
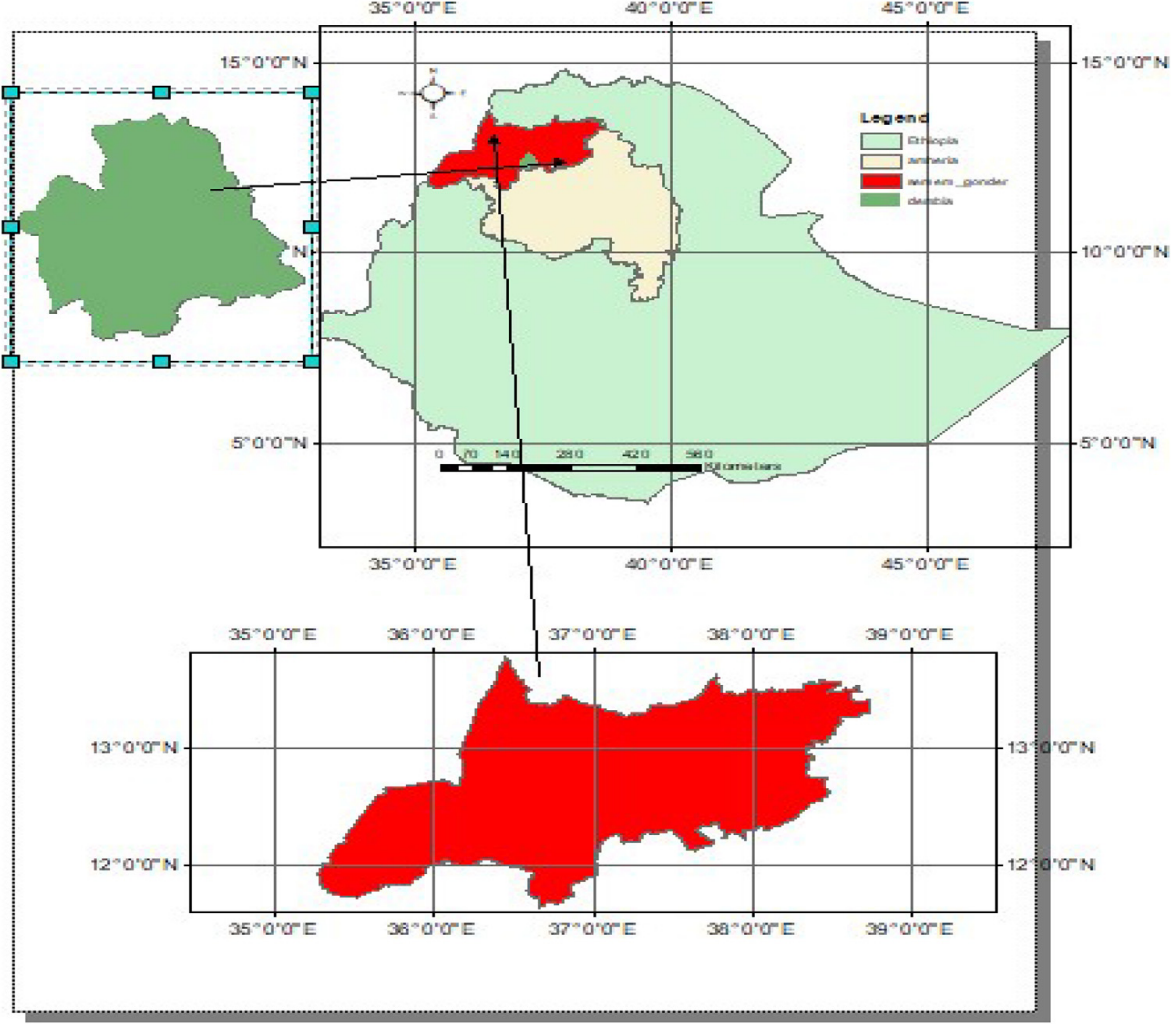
Geographical location of the study area, Gorgora, West Dembia, Ethiopia.

**Fig. 2:**
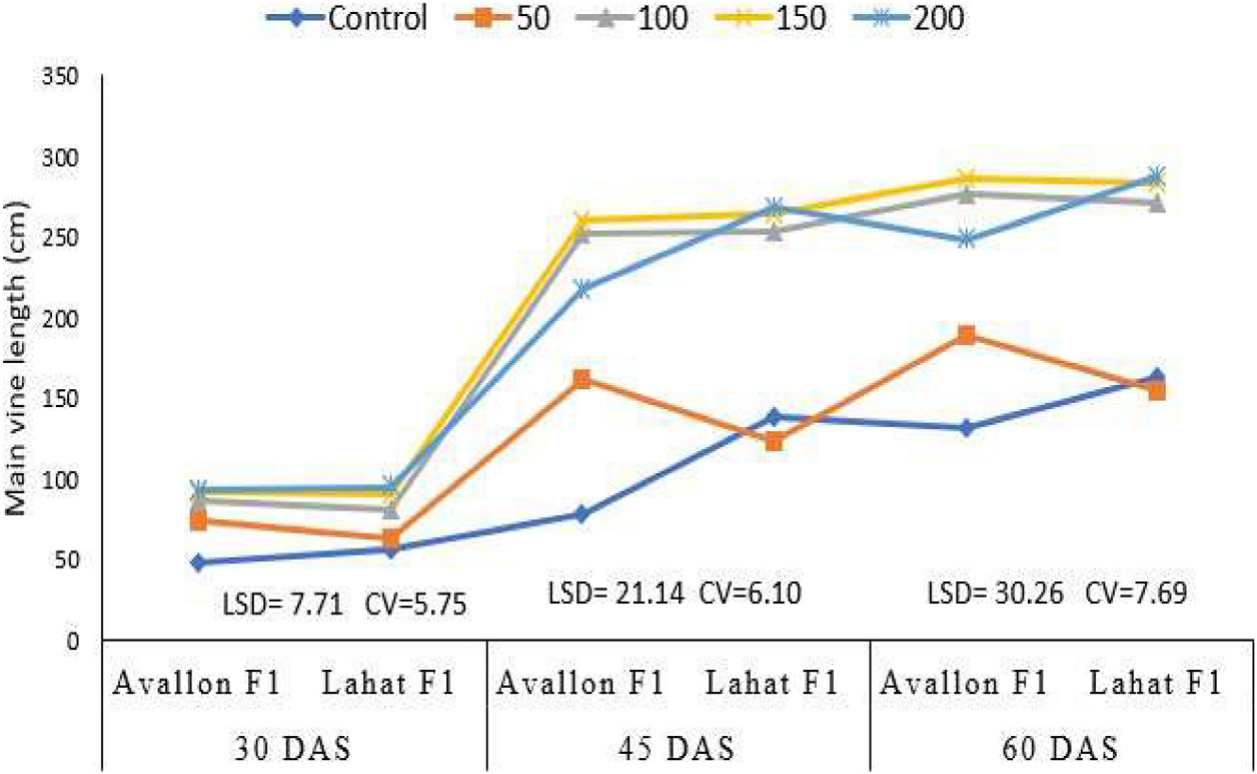
Main vine length of watermelon as affected by interaction effect of K fertilizer rates and varieties. DAS= Days after sowing.

**Fig. 3:**
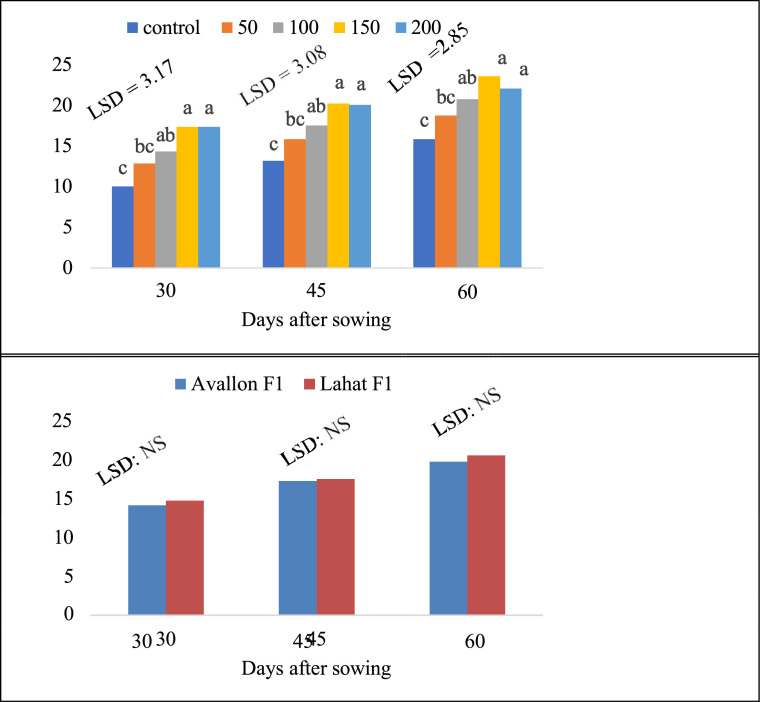
Main effects of K fertilizer rates and varieties on numbers of leaves on main vine of watermelon.

**Fig. 4:**
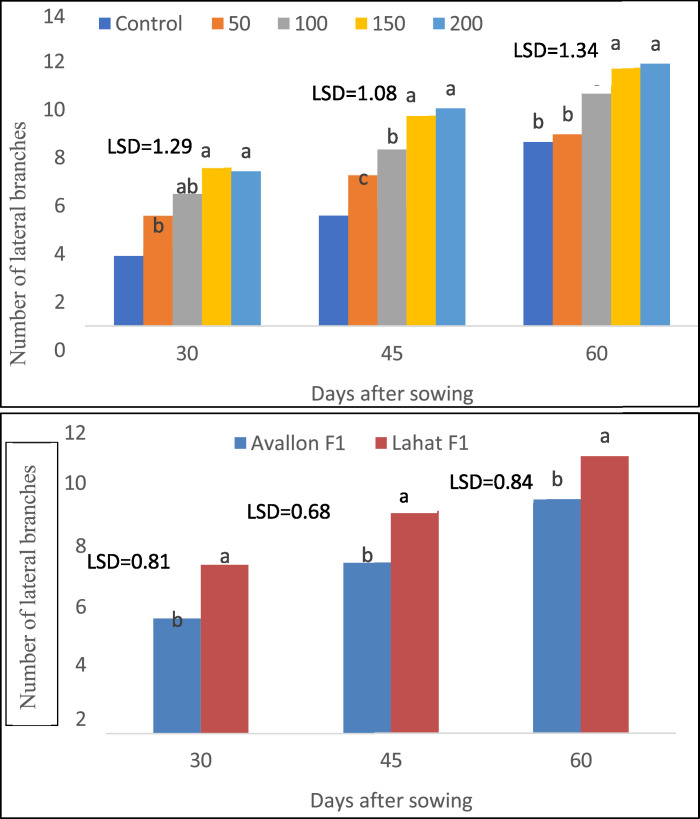
Main effects of K fertilizer rates and varieties on numbers of lateral branches per vine of watermelon.

This dataset was collected from a field experiment conducted at West Dembia District, Central Gondar Zone, Ethiopia, to evaluate the effects of K fertilizer rates on two watermelon (*Citrullus lanatus* [Thunb.]) hybrid varieties, Lahat F1 and Avallon F1 (Fig. 1). The experiment was laid out as a factorial design with three replications under irrigated conditions. The effects of K fertilizer rates on crop phenology parameters, such as emergence days and flowering days and, maturity days are presented in Table 1, Table 2, respectively. The dataset shows how these parameters of watermelon hybrid varieties responded to levels as shown in Fig. 2, Fig. 3 and 4.

**Table 1:** Main effects of K fertilizer rates and varieties on watermelon crop phenology parameters.

Treatments	Days to emergence	Days to 50 % Flowering
K (kg ha-1)		
Control	7.8	54.1^a^
50	9.1	48.8^b^
100	8.3	46.8^c^
150	8.0	42.8^d^
200	9.0	42.0^d^
LSD(0.05)	NS	1.9
Varieties		
Avallon F1	7.3^b^	46.3^b^
Lahat F1	9.6^a^	47.5^a^
LSD(0.05)	0.7	1.1
CV (%)	11.0	3.3

**Table 2:** Maturity days of variety in response to K fertilization.

K rates (kg ha^-1^)	Varieties	
	Avallon F1	Lahat F1
Control	80.5^b^	85.4^a^
50	73.1^e^	77.1^c^
100	72.0^fg^	75.0^d^
150	71.1^gh^	72.8^ef^
200	69.0^i^	71.0^h^
LSD(0.05)	0.9	
CV (%)	0.7	

Means followed by the same letters in a column and rows are not significantly different from each other according to the LSD test at 5 % level of significance.

The main effects included: 1) the performance of hybrid varieties across different K rates and 2) the effect of K rates on hybrid varieties. The interaction effects represented the response of each hybrid variety to varying K fertilizer rates, including main vine length, number of leaves on the main vine, and lateral branches per vine, as shown in Fig. 2, Fig. 3, Fig. 4. These growth indicators highlight the response of watermelon plants to different K fertilizer treatments, with significant differences observed among the fertilizer levels and between the two varieties.

The interaction effects included: 1) the performance of hybrid varieties across different K rates and 2) the effect of K rates on hybrid varieties. The main effects focused on the response of each hybrid variety to varying K fertilizer rates. Parameters such as fruit length, fruit diameter, average fruit weight, marketable yield, unmarketable yield, total yield, and fruit pulp weight are highlighted in Tables 3 and 5. Meanwhile, parameters such as marketable fruits per plant, unmarketable fruits per plant, and total fruits per plant were influenced by the main effects of K fertilizer rates and watermelon varieties, as shown in Table 4.

**Table 3:** Yield and yield component parameters (i) of watermelon as affected by interaction effect of K fertilizer rates and varieties.

Parameters	K fertilizer rates (kg ha^-1^)	Varieties	
		Avallon F1	Lahat F1
	Control	46.0e	45.2e
	50	49.4d	49.6d
	100	50.7c	51.4c
Fruit length	150	53.3b	55.8a
(cm)	200	53.3b	55.3a
	LSD (0.05)	1.0	
	CV (%)	1.1	

	Control	74.3e	73.5e
	50	77.7d	77.9d
	100	79.0c	79.7c
Fruit diameter	150	81.6b	84.1a
(cm)	200	81.6b	83.7a
	LSD (0.05)	1.0	
	CV (%)	0.7	

	Control	6.3e	5.5e
	50	9.7d	9.9d
	100	11.0c	11.7c
Average fruit	150	13.6b	16.1a
weight (kg)	200	13.6b	15.7a
	LSD (0.05)	1.0	
	CV (%)	5.4

**Table 4:** Main effects of K fertilizer rates and varieties on yield and yield component parameters of watermelon.

Treatments	NMFPP	NUMFPP	TNFPP
K rates(kg ha^-1^)			
Control	0.6^d^	0.7	1.4^d^
50	2.8^c^	0.5	3.4^c^
100	3.6^b^	0.5	4.2^b^
150	5.4^a^	0.5	6.0^a^
200	5.2^a^	0.5	5.7^a^
LSD(0.05)	0.4	NS	0.4
Varieties			
Avallon F1	3.3^b^	0.5	3.9^b^
Lahat F1	3.7^a^	0.6	4.4^a^
LSD(0.05)	0.2	NS	0.2
CV (%)	10.7	31.07	8.61

Where: NMFPP is number of marketable fruits plant^-1^, NUMFPP is number of un-marketable fruits plant^-1^, TFPP is total number of fruits plant^-1^.

**Table 5:** Yield parameters of watermelon as affected by interaction effect of K fertilizer rates and varieties.

Parameters	K (kg ha^-1^)	Varieties	
		Avallon F1	Lahat F1
	Control	59.8^e^	59.0^e^
Marketable yield (t ha-1)	50	63.3^d^	63.4^d^
	100	64.5^c^	65.2^c^
	150	67.1^b^	69.6^a^
	200	67.1^b^	69.2^a^
	LSD (0.05)	1.0	
	CV (%)	0.9	

	Control	7.1^a^	7.6^a^
	50	5.5^b^	5.4^bc^
Unmarketable yield (t ha^-1^)	100	4.8^cd^	4.5^d^
	150	3.7^e^	2.4^f^
	200	3.6^e^	2.1^f^
	LSD (0.05)	0.6	
	CV (%)	7.6	

	Control	67.0^g^	66.7^g^
	50	68.8^f^	68.9^ef^
Total yield (t ha^-1^)	100	69.4^de^	69.8^d^
	150	70.9^bc^	72.0^a^
	200	70.7^c^	71.3b
	LSD (0.05)	0.5	
	CV (%)	0.4	

	Control	3.4^h^	3.2^h^
	50	5.2^g^	5.4^f^
Fruit pulp weight (kg)	100	5.8^e^	6.3^d^
	150	7.3^c^	8.5^a^
	200	7.2^c^	8.2^b^
	LSD (0.05)	0.2	
	CV (%)	2.1	

Means followed by the same letters in a column and rows are not significantly different from each other according to the LSD test at 5 % level of significance.

Quality parameters, including soluble solids (°Brix), fruit firmness, shelf life, and titratable acidity, are outlined in Table 6, Table 7. These parameters provide insights into how K fertilizer affected the internal quality of the fruits, such as sweetness, texture, and storage potential (Table 8).

**Table 6:** Main effects of K fertilizer rates and varieties on quality parameters of watermelon.

Treatments	TSS (°Brix)	Fruit firmness (N)	Shelf life (days)
K (kg ha-^1^)			
Control	10.0^c^	6.3^c^	7.7^b^
50	10.3^c^	6.5^c^	8.1^b^
100	10.7^bc^	6.9^bc^	8.5^ab^
150	11.5^ab^	7.6^ab^	9.2^a^
200	12.0^a^	8.2^a^	9.2^a^
LSD(0.05)	0.9	0.2	1.0
Varieties			
Avallon F1	10.3^b^	5.3	8.0
Lahat F1	11.5^a^	5.2	9.1
LSD(0.05)	0.5	NS	0.6
CV (%)	7.0	3.5	9.8

Means followed by the same letters in a column are not significantly different from each other the LSD test at 5 % level of significance.

**Table 7:** Total titratable acidity of watermelon as affected by interaction effect of K fertilizer rates and varieties.

K (kg ha^-1^)	Varieties	
	Avallon F1	Lahat F1
Control	0.18^a^	0.17^b^
50	0.16^c^	0.15^d^
100	0.14^f^	0.15^e^
150	0.14^g^	0.14^f^
200	0.13^h^	0.12^i^
LSD (0.05)	2.78	
CV (%)	0.01	

Means followed by the same letters in a column and rows are not significantly different from each other according to the LSD test at 5 % level of significant.

**Table 8:** Net benefit and marginal rate of return (MRR) analysis of K fertilizer and varieties on watermelon marketable fruit yield.

K rates (kg ha^-1^)	Varieties	MFY (t ha^-1^)	AjMY (t ha^-1^)	GI ETB ha^-1^)	TVC (ETB)	NT (ETB ha^-1^)	MRR (%)
Control	Lahat F1	59.06	53.15	1,594,500	10,000	1,584,500	–
	AvallonF1	59.86	53.87	1,616,100	12,000	1,604,100	980
50	Lahat F1	63.46	57.11	1,713,300	14,750	1,698,550	3434.54
	AvallonF1	63.30	56.97	1,709,100	15,250	1,693,850	D
100	Lahat F1	65.26	58.73	1,761,900	16,500	1,745,400	2677
	AvallonF1	64.56	58.10	1,743,000	18,500	1,724,500	D
150	Lahat F1	69.60	62.64	1,879,200	19,750	1,859,450	3509.23
	AvallonF1	67.13	60.42	1,812,600	21,750	1,790,850	D
200	Lahat F1	69.20	62.28	1,868,400	23,000	1,845,400	D
	AvallonF1	67.13	60.42	1,812,600	25,000	1,787,600	D

MFY= Marketable fruit yield; AjMY= Adjusted marketable yield; GI= Gross income; TVC= Total variable cost; NT= Net benefit; MRR= Marginal rate of return; *D*= Dominated.

Overall, the dataset reveals that potassium fertilizer significantly influenced both the growth and yield performance of the watermelon varieties, with higher rates of potassium generally leading to improved yield and fruit quality. The interaction between fertilizer rates and varieties played a crucial role in determining the optimal nutrient levels for maximizing both productivity and fruit quality in watermelon production under field conditions.

## 4 Experimental Materials, Design and Methods

This experiment was conducted to evaluate the effects of different potassium fertilizer levels on the growth, yield, and quality of two watermelon varieties (Citrullus lanatus [Thunb.]), Lahat F1 and Avallon F1, under field conditions. The study took place at Gorgora, West Dembia, Ethiopia, on soil characterized as sandy loam with a pH of 6.5, organic matter content of 1.2 %, and available potassium of 112 mg/kg. These properties were determined prior to the experiment to provide baseline soil fertility conditions. Both varieties were selected for their high market potential and adaptability to the local climate, with seeds sourced from the market.

The experiment consisted of 30 plots, each measuring 3 m × 2.5 m. Plants were spaced 1.6 m apart between rows and 0.75 m between plants within each row to provide sufficient room for vine growth and ensure good air circulation, thereby minimizing the risk of disease. To prevent interference between treatments, a 1.5 m gap was maintained between blocks, and a 1 m gap was left between plots within each block.

KCl fertilizer was applied at planting time. In addition to potassium, nitrogen was supplied as urea (46 % N) at the rate of 100 kg/ha, split into two equal applications: 50 % at planting time and the remaining 50 % applied 30 days after emergence. Phosphorus, provided as Di-Ammonium Phosphate (DAP) at a rate of 100 kg/ha, was applied at planting time.

Watermelon plants were irrigated using a surface irrigation system. During the establishment phase, irrigation was applied every 3 days to ensure uniform germination and healthy early growth. Once the plants were well established, irrigation frequency was adjusted to 7-day intervals, ensuring that the soil moisture was maintained at field capacity, which is crucial for optimal growth and fruit development.

Weed management was carried out manually using hand weeding every two weeks throughout the growing season to minimize competition for nutrients, water, and sunlight. For pest and disease management, an integrated approach was employed. Aphid infestations were controlled by applying an insecticidal soap (at a rate of 2 % solution) every three weeks, while fungal diseases such as powdery mildew were managed using a fungicide containing sulfur (applied at 10-day intervals). Regular field monitoring was conducted to identify any emerging pest or disease issues, and appropriate corrective measures were taken based on standard watermelon cultivation practices

Harvesting took place when the fruits reached physiological maturity, which was identified by visual indicators such as the drying of the tendrils closest to the fruit and the characteristic hollow sound produced when tapping the fruit. Fruit quality parameters, including fruit weight, pulp weight, fruit length, fruit diameter, and soluble solids (°Brix), were measured during the harvest to assess the effects of the potassium fertilizer treatments

## 5 Data Collected

### 5.1 Phenology parameters

Key phenological data were collected throughout the watermelon growth cycle. Days to emergence were recorded as the number of days from sowing until 50 % of the seedlings had emerged. Days to flowering were measured from sowing to the point when 50 % of the plants exhibited fully developed flowers, marking the onset of reproductive growth. Days to maturity were documented when the fruits reached physiological maturity, indicated by the drying of tendrils closest to the fruit, the change in fruit rind color, and the characteristic hollow sound produced when tapping the fruit. These phenology parameters provided insights into the developmental timeline of the watermelon varieties under varying potassium treatments.

### 5.2 Growth parameters

Growth measurements focused on the vegetative performance of the watermelon plants. Main vine length (cm) was measured at two critical growth stages: fruit set and physiological maturity. The length was recorded from the base of the plant to the tip of the main vine on ten randomly selected plants per plot. Number of leaves per vine was counted on the same ten plants to evaluate foliage development, which plays a key role in photosynthesis and overall plant health. The number of lateral branches per vine was also recorded, providing additional data on the branching pattern, which is crucial for supporting fruit development and maximizing yield potential.

### 5.3 Yield and yield component parameters

Yield data were central to evaluating the effects of potassium fertilizer on watermelon production. Marketable fruits per plant were counted based on size, shape, and the absence of defects such as cracks, malformation, or disease. Only fruits meeting commercial standards were classified as marketable. Unmarketable fruits per plant were those showing defects, including undersized or deformed fruits. The total number of fruits per plant was the sum of both marketable and unmarketable fruits, reflecting the overall fruiting capacity of the plants.

Total yield (t/ha) was calculated by weighing all harvested fruits from each plot and converting the data into tons per hectare. Average fruit weight (kg) was derived by weighing 10 randomly selected fruits from each plot and calculating the mean. In addition, pulp weight (kg) was measured by separating and weighing the edible flesh of the selected fruits, providing an assessment of the internal fruit quality. Fruit length (cm) and fruit diameter (cm) were measured using a measuring tape and Vernier caliper, respectively, to evaluate the physical dimensions of the fruits, both of which are important quality traits for market preference.

### 5.4 Quality parameters

A range of fruit quality parameters was assessed to understand the influence of potassium on the internal and external quality of the watermelons. Soluble solids content (°Brix), a key indicator of sweetness and consumer appeal, was measured using a refractometer. Fruit firmness (N) was determined using a penetrometer to assess the texture and resistance of the fruit flesh, an important quality characteristic for both shelf life and transport. The shelf life (days) was evaluated by monitoring the number of days fruits remained fresh under ambient storage conditions, providing insights into post-harvest performance. Titratable acidity (%), which influences the fruit's flavor profile, was measured by titrating the juice to determine the percentage of organic acids present in the fruit.

### 5.5 Statistical data analysis

Data were subjected to Analysis of Variance (ANOVA**)** using R software version 4.2. The analysis was performed utilizing general linear model (GLM) procedures to assess the effects of different potassium fertilizer levels and watermelon varieties on various growth, yield, and quality parameters. Treatment means were separated using the Least Significant Difference (LSD) test at the 1 % or 5 % level of significance, depending on the outcomes of the ANOVA, ensuring robust comparisons among treatment groups.

In addition, correlation analysis was conducted to evaluate relationships between growth and yield parameters. Simple linear correlation coefficients were calculated to determine the strength and direction of associations between variables, enabling insights into how various growth metrics influence overall fruit yield and quality.

### 5.6 Partial budget analysis

An economic analysis was conducted to assess the feasibility of potassium fertilizer treatments on watermelon production. The analysis employed partial budget, dominance, and marginal analysis methods to evaluate the economic viability of the treatments. A partial budget was utilized to systematically organize experimental data and information regarding the costs and benefits associated with each potassium fertilizer treatment.

The partial budget was calculated using adjusted marketable yield data. The total variable costs associated with each treatment included the costs of potassium fertilizer, nitrogen (urea), and their application. The costs were determined as follows: potassium fertilizer at Birr 20 per kg, urea at Birr 15 per kg, and application costs at Birr 0.5 per kg Transportation costs for the harvested watermelons were considered at Birr 0.1 per kg.

To account for potential losses, the marketable yield was adjusted downward by 10 %, based on the assumption that farmers might experience a reduction in yield due to various cultivation challenges (CIMMYT, 1988). The selling price for watermelon was estimated at Birr 30.00 per kg in 2021, which was used for revenue calculations.

Any treatment that yielded net benefits less than or equal to those of a less costly treatment was classified as dominant and excluded from marginal analysis. It is generally accepted that the minimum rate of return acceptable to farmers ranges between 50 % and 100 % (CIMMYT, 1988). Consequently, a minimum acceptable marginal rate of return of 100 % was adopted for this study.

This partial budget analysis highlights the economic implications of using potassium fertilizer in watermelon production, providing critical insights for farmers looking to enhance their profitability and yield.

## Limitations

One limitation of the data is that it was collected from a single location (Gorgora) over one growing season, which may limit the generalizability of the findings to other regions or seasons. Variations in climate, soil conditions, and environmental factors in different locations could affect the response of watermelon varieties to potassium fertilizer rates. Additionally, the dataset is specific to two hybrid watermelon varieties, which may not fully represent the broader genetic diversity of watermelons. Future studies conducted across multiple locations and over several growing seasons could help address these limitations and provide a more comprehensive understanding

## Ethical Statement

The authors confirm that they have read and adhere to the ethical requirements for publication in Data in Brief. The current work does not involve human subjects, animal experiments, or any data collected from social media platforms. All research practices followed ethical guidelines and standards relevant to data collection and analysis.

## CRediT Authorship Contribution Statement

**Conceptualization**: Hulushum Woreta Kassa - Developed the research idea and formulated the study design, including the selection of hybrid watermelon varieties and potassium fertilizer rates. **Methodology**: Hulushum Woreta Kassa - Designed the experimental setup and determined the data collection methods. Asrat Ayalew Teka - Assisted in refining the methodology and ensuring adherence to agronomic standards. **Data Collection**: Hulushum Woreta Kassa - Conducted field experiments and collected data on crop phenology, growth, yield, and quality parameters. Aleminew Tagele Dellele - Supported data collection efforts and assisted with measurements. **Data Analysis**: Hulushum Woreta Kassa - Performed statistical analysis of the data using R software and interpreted the results. Abebaw Mulugeta Andualem - Contributed to the validation of analysis methods and discussed implications. **Writing – Original Draft**: Hulushum Woreta Kassa - Wrote the initial draft of the manuscript. **Writing – Review & Editing**: Hulushum Woreta Kassa, Asrat Ayalew Teka, Aleminew Tagele Dellele, and Abebaw Mulugeta Andualem - Reviewed and edited the manuscript for clarity, coherence, and compliance with journal guidelines

## Data Availability

Mendeley Datawatermelon data for publication (Original data).
